# The characteristic and potential therapeutic effect of isolated multidrug-resistant *Acinetobacter baumannii* lytic phage

**DOI:** 10.1186/s12941-022-00492-9

**Published:** 2022-01-07

**Authors:** Behnam Sisakhtpour, Arezoo Mirzaei, Vajihe Karbasizadeh, Nafiseh Hosseini, Mehdi Shabani, Sharareh Moghim

**Affiliations:** Department of Bacteriology and Virology, Faculty of Medicine, Isfahan University of Medical Sciences, 81744-176 Isfahan, Iran

**Keywords:** Multi-drug resistant *Acinetobacter baumannii*, Bacteriophage, Phage therapy, Endolysin activity

## Abstract

**Background:**

Widespread misuse of antibiotics caused bacterial resistance increasingly become a serious threat. Bacteriophage therapy promises alternative treatment strategies for combatting drug-resistant bacterial infections. In this study, we isolated and characterized a novel, potent lytic bacteriophage against multi-drug resistant (MDR) *Acinetobacter baumannii* and described the lytic capability and endolysin activity of the phage to evaluate the potential in phage therapy.

**Methods:**

A novel phage, pIsf-AB02, was isolated from hospital sewage. The morphological analysis, its host range, growth characteristics, stability under various conditions, genomic restriction pattern were systematically investigated. The protein pattern of the phage was analyzed, and the endolysin activity of the phage was determined under the non-denaturing condition on SDS-PAGE. The optimal lytic titer of phage was assessed by co-culture of the phage with clinical MDR *A. baumannii* isolates. Finally, HeLa cells were used to examine the safety of the phage.

**Results:**

The morphological analysis revealed that the pIsf-AB02 phage displays morphology resembling the *Myoviridae* family. It can quickly destroy 56.3% (27/48) of clinical MDR *A. baumannii* isolates. This virulent phage could decrease the bacterial host cells (from 10^8^ CFU/ml to 10^3^ CFU/ml) in 30 min. The optimum stability of the phage was observed at 37 °C. pH 7 is the most suitable condition to maintain phage stability. The 15 kDa protein encoded by pIsf-AB02 was detected to have endolysin activity. pIsf-AB02 did not show cytotoxicity to HeLa cells, and it can save HeLa cells from *A. baumannii* infection.

**Conclusion:**

In this study, we isolated a novel lytic MDR *A. baumannii* bacteriophage, pIsf-AB02. This phage showed suitable stability at different temperatures and pHs, and demonstrated potent in vitro endolysin activity. pIsf-AB02 may be a good candidate as a therapeutic agent to control nosocomial infections caused by MDR *A. baumannii*.

**Supplementary Information:**

The online version contains supplementary material available at 10.1186/s12941-022-00492-9.

## Background

*Acinetobacter baumannii* (*A. baumannii*) is responsible for many health care infections, particularly burn and wound infections [1]. This non-fermentative, non-motile, and aerobic gram-negative bacterium is listed as one of the six most dangerous pathogens, namely ESKAPE (*Enterococcus faecium, Staphylococcus aureus, Klebsiella pneumonia, Acinetobacter baumannii, Pseudomonas aeruginosa,* and *Enterobacter* spp.) [2]. ESKAPE pathogens are resistant to antibiotics and are responsible for the majority of nosocomial infections [2, 3]. Recently, some strains of *A. baumannii* were found to be resistant to nearly all known antibiotics [4, 5]. Multidrug-resistant (MDR) *A. baumannii* refers to *A. baumannii* strains resistant to at least three of the five types of antimicrobial agents, including β-lactamase inhibitors, carbapenems, cephalosporins, fluoroquinolones, and aminoglycosides [6]. Therefore, alternative treatments for these infections are urgently needed.

Bacteriophage therapy is a promising alternative treatment for MDR bacterial infections. A bacteriophage (phage) is a virus which infects and lyses the bacterial host. Phage therapy is a century-old therapeutic method applied for the treatment of bacterial infections [7, 8]. With the increasing emergence of antimicrobial resistance, the focus on phage therapy has been renewed [9]. The phages employed for therapy display many advantages, including host specificity (do not affect normal flora and eukaryotic cells), rapid replication inside the bacteria, and killing the host cells [10–12]. In addition to the application of lytic phages in the treatment of bacterial infections, phage-derived antimicrobial substances, such as endolysin, are identified as potent antimicrobial agents and have been utilized as a successful treatment for the bacterial infections in vitro and in animal models [13]. Thus, the isolation and characterization of lytic phages is a potential strategy for fighting Multi-Drug Resistance (MDR) *A. baumannii* [11, 14]. Prior to clinical application, potential therapeutic phages must be thoroughly examined for safety and effectiveness [15, 16].

## Materials and methods

### Bacterial isolation and identification

This study included 48 clinical isolates of *A. baumannii*. All clinical samples were taken from patients admitted to Intensive Care units (ICUs) at the Medical University hospitals of Isfahan, Iran, during 2016–2018. All specimens were cultured initially on blood and MacConkey agar (Merck) and incubated for 24 h at 37 °C. Clinical isolates were identified based on conventional microbiological methods [17] and confirmed by PCR. The genomic DNA of the bacterial isolates were extracted by boiling method, as described by Dashti et al. [18]. PCR was performed based on the amplification of the *blaOXA-51* gene for the molecular identification of *A. baumannii* isolates. The PCR condition and the Primers used in this study were defined previously [19].

### Antibiotic susceptibility testing

Agar disk diffusion method was performed to determine the susceptibility of the isolates to various antibiotics, including amikacin (30 μg), cefepime (30 μg), ceftazidime (30 μg), ciprofloxacin (5 μg), and rifampin (5 μg), (Rosco, Denmark). The inhibition of bacterial growth was measured and compared to the reference tables provided by the Clinical and Laboratory Standards Institute (CLSI 2018) [20].

### Isolation, purification, and titration of lytic phages

For isolating phages, sewage samples were collected from various water sources in Alzahra General Hospital (Isfahan, Iran). A clinical MDR *A. baumannii* (MDR-AB02) was used as an indicator for bacteriophage screening of the sewage samples. The phages were isolated and enriched using the enrichment method [21]. Briefly, 50 ml of centrifuged sewage supernatant was filtered through a 0.45 µm pore size membrane and mixed with an equal volume of 2 × nutrient broth containing 1 ml exponential phase of MDR *A. baumannii* (OD_600_ = 0.6) to enrich the phages at 35 °C overnight with shaking at 160 rpm. The culture was centrifuged for 10 min at 13,000×*g* rpm, and then the supernatant was filtered through a 0.45 µm pore-size membrane filter to remove the residual bacteria. Subsequently, 200 µl of the filtrate was mixed with 100 µl of the MDR *A. baumannii* (OD_600_ = 0.6) and 2.5 ml of soft nutrient agar (0.7% agar). Then, the mixture was overlayed onto a solidified nutrient agar (1.5% agar) and incubated for 24 h at 37 °C. The clear plaques were picked, and a double-layer agar method was performed to obtain purified phage. Each individual phage was purified by several rounds of plaque picking, and the purification process was repeated until single-plaque morphology was observed [22]. The phage titer was determined by the double-layer agar method, and the titer was reported as a plaque-forming unit (PFU/ml) [23].

### Phage concentration and storage

Each single purified plaque was added into 5 ml of nutrient broth containing the MDR*-*AB02 (OD_600_ = 0.6) and cultured at 37 °C for 24 h. Then, the suspension was transferred into 500 ml of nutrient broth and shaken overnight at 35 °C. Chloroform was added to a final concentration of 0.1%, mixed gently, and allowed to stand at room temperature for 15 min to kill the bacteria. Solid NaCl was added to the culture to a final concentration of 1 M, mixed and dissolved, and the culture was incubated in an ice bath for one hour. In order to remove cell debris, centrifugation at 10,000×*g* for 10 min was done, and solid PEG6000 was added to the supernatant to a final concentration of 10% (w/v) while mixed and dissolved slowly at room temperature. The solution was incubated for 1 h on ice to precipitate the phage particles. After centrifugation (10,000×*g*) for 10 min at 4 °C, the pellet was suspended in 5 ml of SM buffer (50 mM Tris–Cl, 100 mM NaCl, 8 mM MgSO4, pH 7.5) [45]. An equal volume of chloroform was then added to separate the phage particles from PEG6000. After centrifugation at 3000×*g* for 10 min, the supernatant was passed through a 0.22 μm pore-size membrane filter and stored at 4 °C [24].

### Examination of the phage morphology by transmission electron microscopy (TEM)

A drop of phage solution was placed onto a copper mesh grid surface and negatively stained with 2% phosphotungstic acid (PTA). The grid was examined by transmission electron microscopy (Zeiss–EM10c, Germany) at an operating voltage of 100 kV.

### pH, thermal, and chloroform stability

For the pH stability test, 10^10^ PFU/ml of the phage aliquots were treated with various pH buffers (3, 5, 7, 9, and 11) at 37 °C in SM buffer for 1 h. The phage titer was determined by the double-layer agar method, as described above. As for the thermal stability, the phage preparations were incubated at pH 7 in SM buffer at different temperatures (37 °C, 50 °C, and 70 °C) for one hour, and the titer of the virus was assessed. To determine chloroform stability, 1 ml (1 × 10^10^ PFU) of the phage was mixed with 0.4 ml chloroform, and the phage was collected and titered after one hour incubation at room temperature [25].

### Determination of optimal phage titer

To decrease the bacterial concentration, the optimal titer of the phage was determined. An overnight culture of MDR *A. baumannii* was transferred to 30 ml of nutrient broth medium grown at 35℃ until the OD_600_ of the culture reached 1.0. A serial dilution of the isolated phage (10^6^–10^9^ PFU/ml), equal to Multiplicity of infection (MOI) of 0.01, 0.1, 1, and 10, was prepared and inoculated to the fresh MDR *A. baumannii* culture, separately. The mixtures were incubated at 35℃. One milliliter of the culture sample was removed at interval time and centrifuged at 12,000 for 5 min to separate the pellet from the supernatant. Then the bacterium pellet was washed with phosphate buffer saline (PBS) and resuspended in 1 ml PBS. The bacterial suspension was serial diluted and spread on the nutrient agar (1.5%). The titer was assessed by counting the visible bacteria on the plate and represented as a colony-forming unit (CFU/ml) [26].

### One-step growth curve

For the one-step growth curve experiment, one milliliter of the MDR *A. baumannii* suspension at Nutrient Broth (OD600 = 0.1) in the exponential phase was mixed with the phage with a final concentration of 10^6^ PFU/ml at an MOI 0.01 and let to adsorb for 10 min. The unabsorbed phages were removed by brief centrifugation (6000*g*, 10 min), and 50 µl of the pellet was transferred to 50 ml of Nutrient Broth medium and placed at 37° C on a shaker (160 rpm). Samples were collected every 10 min over a time period of 120 min, and the number of phages was immediately assessed by the double-layer agar method [26]. This experiment was done in triplicate.

### Phage genome analysis with restriction enzymes

The phage DNA was extracted using the Viral Nucleic Extraction Kit II (Geneaid, Taipei, Taiwan). The phage DNA was digested with the *HindIII*, *HincII*, *EcoRI,* and *NheI* restriction enzymes (Sigma Aldrich) according to the manufacturer's protocol. Restriction digestions were repeated three times. The digested DNA was analyzed by 0.8% agarose gel electrophoresis with 0.5% TBE (Tris–Borate EDTA) running buffer [26].

### Phage protein analysis under denaturing conditions

For protein analysis, precipitated purified phage particles were denatured in loading buffer (50 mM Tris–HCl, 1% β-Mercaptoethanol, 2% sodium dodecyl sulfate (SDS), 10% glycerol, and 0.1% bromophenol blue). Samples were heated in a boiling water bath for 3 min and subjected to SDS-PAGE. The separated protein bands were visualized by the coomassie Blue G-250 staining method [27].

### Phage protein analysis under non-denaturing conditions

In order to study the lysis protein of the phage, we used SDS-PAGE under non-denaturing conditions [28]. Phage lysates were centrifuged at 13,000×*g* for 30 min at 4 °C. Then, the supernatant was filtered through a 0.22 μm filter and concentrated by ultracentrifugal filtration (Amiqon, Millipore Sigma-Aldrich, USA) according to the manufacturer’s protocol. The concentrated protein sample was mixed with protein loading buffer without β-mercaptoethanol. The samples were then loaded on an SDS-PAGE without boiling. The resolved gel was placed onto an agar-coated plate, in which soft agar mixed with the MDR AB was previously poured onto the gel and incubated at 35℃ overnight. Clear zones on the overlay indicate endolytic activity.

### Bacteriophage host range

The phage host range was evaluated by the spot method. In brief, 43 MDR *A. baumannii* clinical isolates, *P. aeruginosa* (ATCC 27853), *E. coli* (ATCC 25922), *K. pneumonia* (ATCC 10031) were included for the determination of the lytic spectrum of the isolated phage. Briefly, 200 µl of 10^8^ CFU/ml of each overnight culture of bacteria was mixed separately with 3 ml of 0.6% melted agar (50 °C) and poured onto a solidified nutrient agar coated plate (1.5% agar). After agar was solidified, 10 µl of the filtered phage was spotted on each plate, with *A. baumannii* clinical isolates. The appearance of lysis plaques was investigated after 12 h [29].

### Bacterial reduction assay

We used the method previously described by Ghajavand et al. [30]. Briefly, 1 ml of fresh culture of MDR-AB02 (OD600 = 0.1) was inoculated to two separated flasks containing 100 ml nutrient broth. One flask was inoculated with the isolated phage, and the other one was taken without phage as a negative control. The cultures were incubated at 35 °C at 160 rpm. The optical density (OD _600_) of samples was measured at 20 min intervals for 4 h.

### Cells survival assay

We investigated the toxicity of the isolated phage to Hela cells. HeLa cell line (ATCC CCL-2) was obtained from the National Cell Bank of Iran, Pasteur Institute of Iran (Tehran, I.R. Iran). The HeLa cells (0.5 × 10^4^ cells /well) were seeded in a 96-well cell culture plate in the presence of 100 µl Dulbecco's modified eagle's medium (DMEM, Gibco, USA) supplemented with 5% fetal calf serum (FCS; Gibco, USA), and incubated for 12 h (37 °C in 5% CO2) [15]. Then, 10^6^ CFU/ml of *A. baumannii* (AB02) was added to each well, followed by adding the phage at different MOI (0.01, 0.1, 1, 10). As a control, Hela cells were treated with 10^8^ PFU/ml of the phage without the addition of *A. baumannii*. In a separate experiment, the cells were first infected with 10^6^ CFU of AB02. After 2 h, the phage was added to the infected wells. After incubation for 24 h, the cells were washed twice with PBS, incubated with trypsin solution (0.05% trypsin, 0.5 mM EDTA), and the number of living HeLa cells was counted using Neobar cell count and microscopic observation [26].

## Results

### Bacterial isolates identification and antibiotic susceptibility

**Fig. 1:**
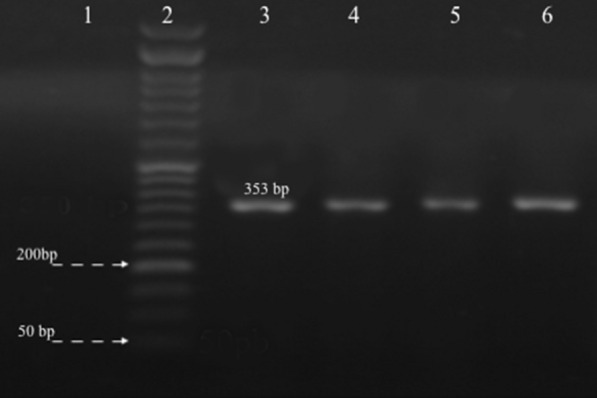
PCR amplification of *bla*_OXA-51_ gene. Lane 1: Negative control, Lane 2: size marker 50 bp, Lane 3: Positive control *A. baumannii* ATCC 19,606, and Lanes 4–6: clinical isolates show bands of amplified DNA at 353 bp

**Fig. 2:**
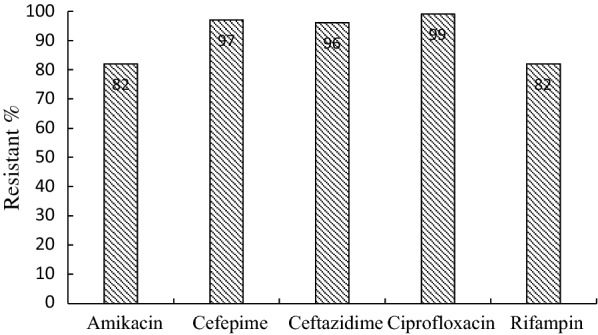
Antimicrobial resistance pattern of *Acinetobacter baumannii* clinical isolates

All 48 clinical samples, which identified *A. baumannii* phenotypically, harbored the *bla*_OXA-51_ gene (Fig. 1). Based on agar disk diffusion assay, 82% of the isolates showed resistance to amikacin, 97% to cefepime, 96% to ceftazidime, 99% to ciprofloxacin, and 82% to rifampin. Few samples had the intermediate resistance pattern, while susceptibility was not found among the MDR *A. baumannii* isolates (Fig. 2).

### Isolation, purification and titration of lytic phages

**Fig. 3:**
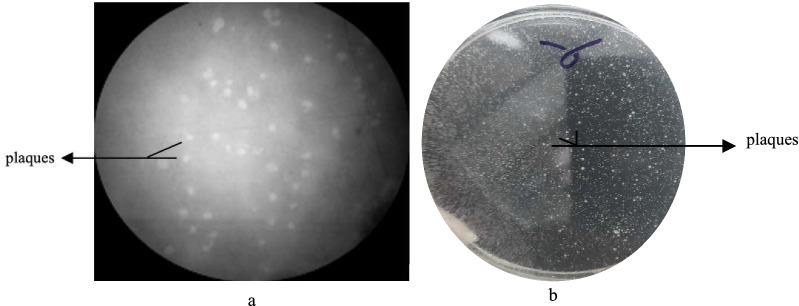
**a** Plaque morphology of phage pIsf-AB02 plaques under light microscope, **b** the clear zone, which demonstrated phage plaque in double-layer agar

MDR *A. baumannii* strain, MDR-AB02, isolated from a patient's catheter with pneumonia at Alzahra hospital, was resistant to more than three groups of antibiotics (Additional file 1: Table S1). This (MDR-AB02) was used as an indicator to screen bacteriophages in sewage samples of the same hospital. The isolated phage was labeled as pIsf-AB02. The pIsf-AB02 forms clear, round, 2–3 mm plaques in the double-layer agar, indicating the lytic property of the phage (Fig. 3). Most MDR *A. baumannii* isolates in this study were sensitive to pIsf-AB02 (27/48); therefore, it was chosen for further study.

### Examination phage morphology by TEM

**Fig. 4:**
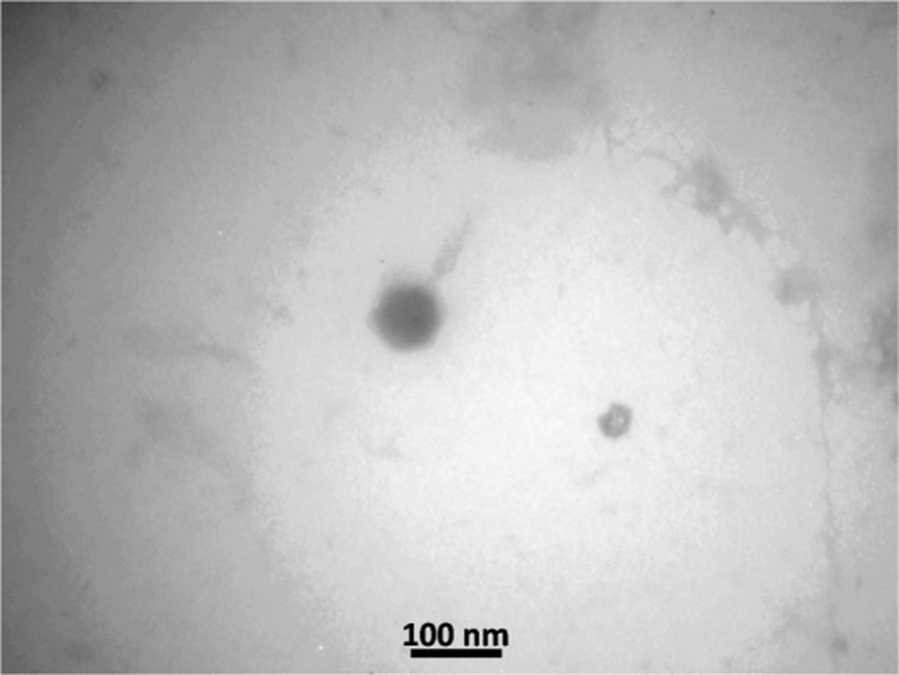
Transmission Electron Microscopy of pIsf-AB02 phage. The bar represents a length of 100 nm

The morphology of pIsf-AB02 was examined by negative staining of the phage and observation under electron microscopy. The phage had an icosahedral head of 70 ± 10 nm and a tail of about 60 nm (Fig. 4). The phage belongs to the order *Caudovirales* and family *Myoviridae* following the current guidelines of the ICTV (International Committee on Taxonomy of Viruses, http://ictv.global/taxonomyRelease.s.asp).

### pH, thermal, and chloroform stability

The stability of the pIsf-AB02 to different pH, chloroform, and temperature was tested. The phage pIsf-AB02 lost its infectivity at pH 3 and 11, while pH 7 is the most suitable condition to maintain the phage. The phage was stable at different temperatures ranging from − 20 to 25 °C. However, the phage titer was slightly dropped at 50 °C and reduced dramatically at 70 °C. The activity of the virus was not affected by chloroform treatment (Additional file 1: Figs. S1, S2).

### Determination of optimal MOI

**Fig. 5:**
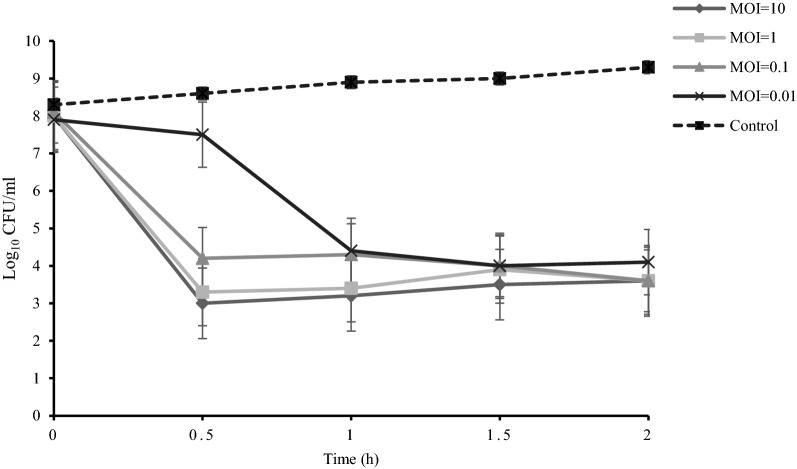
determination of optimal phage titer. The pIsf-AB02 was used at different titers to infect MDR-AB02 to determine the optimal titer of the host during 2 h

The lytic activity of pIsf-AB02 was assessed by inoculating the phage to MDR-AB02. Different MOIs of the phage were inoculated into AB02 (10^8^ CFU/ml). As shown in Fig. 5, The pIsf-AB02 with MOI of 1 reduces the MDR-AB02 from 10^8^ CFU/ml to 10^3^ CFU/ml in 30 min. Lower MOIs (0.1 and 0.01) decreased the virus titer to the same point in 1.5–2 h. The results indicate that although higher MOI reduced *A. baumannii* concentration more quickly, but is not necessary for lysis.

### One-step growth curve

**Fig. 6:**
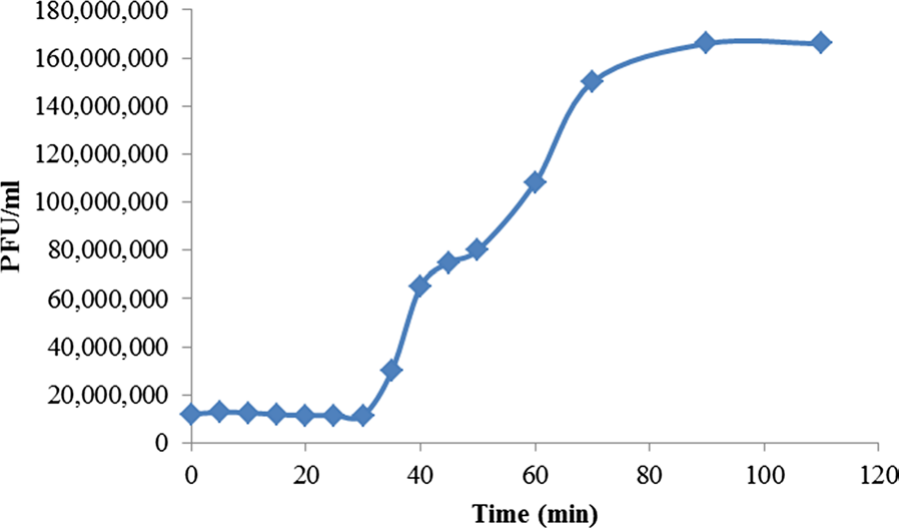
One-step growth curve of pIsf-AB02 phage

One-step growth experiment showed that the latent period of pIsf-AB02 was about 30 min and was followed by the lysis phase, which lasted for 70 min. The burst size was 120 PFU per infected cell (Fig. 6).

### Phage genome analysis

**Fig. 7:**
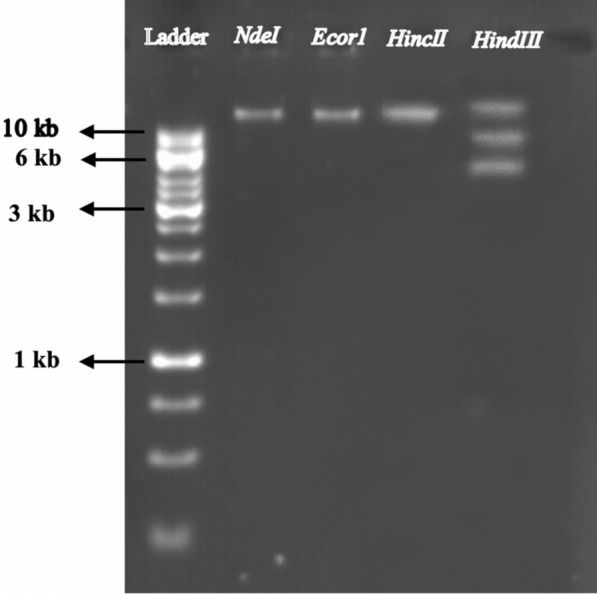
The pIsf-AB02 phage genomic DNA restriction patterns and size determination

The genome analysis indicated that phage pIsf-AB02 has a double-stranded DNA genome (approximately 12.6 kb). The genome of pIsf-AB02 could be digested by *HindIII* endonuclease (Fig. 7). It was found that *HindIII* has three cutting sites. Although, endonucleases, *HincII*, *EcoR1*and *NdeI* have no cutting site.

### Phage protein analysis

**Fig. 8:**
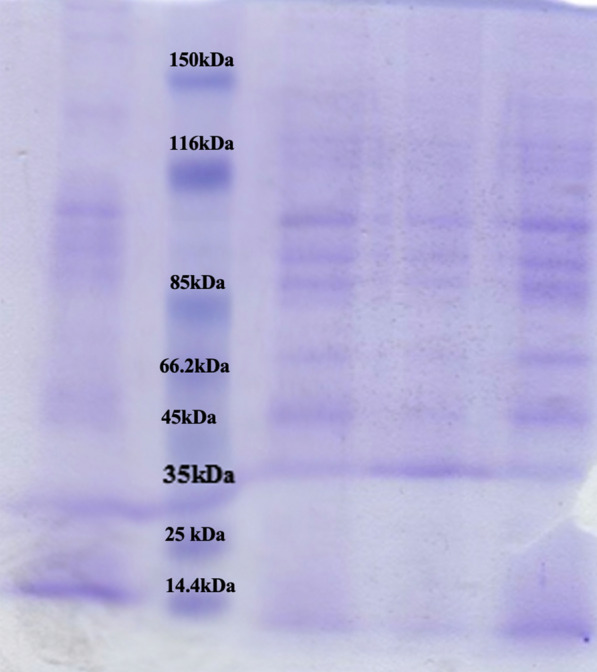
The pIsf-AB02 phage proteins separated by 12% SDS PAGE. Lane 1,4: the phage proteins without boiling, Lane 3,5: the phage proteins with boiling, Lane 2: Ladder

The results of pIsf-AB02 phage protein analysis in denaturing conditions showed nine structural protein bands in 12% SDS-PAGE, with a molecular weight ranging from 14.5 to 150 kDa. The most abundant proteins band in the gel were 100 kDa and 15 kDa. The major band was assumed to be the phage putative coat protein. The latter was predicted to be endolysin, which was confirmed with a non-denaturing condition (Fig. 8).

### Phage protein analysis under non-denaturing conditions (endolysin activity)

**Fig. 9:**
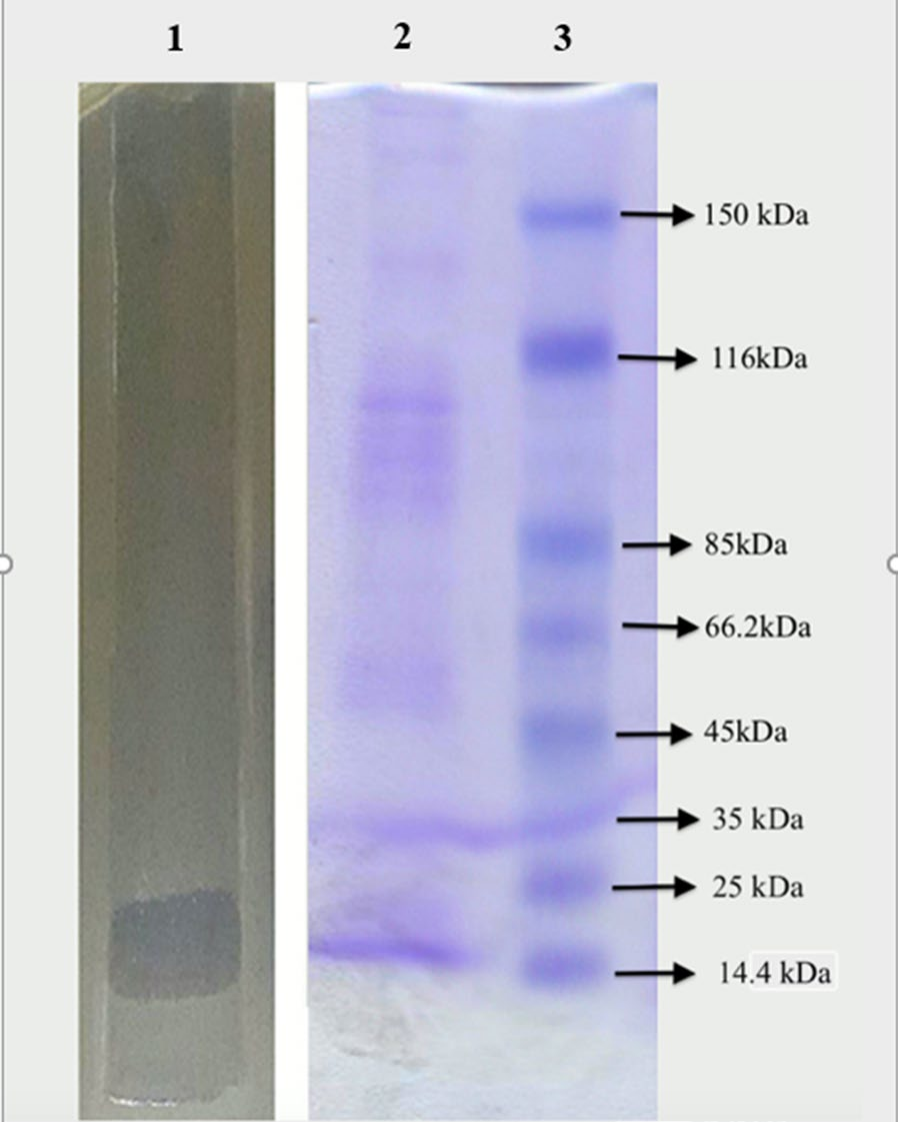
Phage endolysin activity in SDS-PAGE with the non-denaturing condition. The protein from the phage lysate was mixed with sample buffer without β-mercaptoethanol and subjected to SDS-PAGE without boiling. The SDS-PAGE gel was placed onto an agar-containing plate, and soft agar mixed with MDR-AB02 was overlaid. The protein with endolysin activity produced a clear region on the overlay. Lane 1: PAGE overlay on a plate covered by *Acinetobacter baumannii*. Lane 2: phage proteins without boiling in PAGE. Lane 3: protein Ladder

At the time of release, the phages erupt the bacteria, causing the endolysin to flow out into the medium. Proteins of supernatant were concentrated and separated by SDS- PAGE under the non-denaturing condition as described before. The MDR-AB02 overlay on SDS-PAGE showed a clear band at 15 kDa (Fig. 9).

### Bacteriophage host range

Host range spectrum surveyed on forty-eight *A. baumannii* clinical isolates and showed that the pIsf-AB02 phage could infect and lyse 56.3% of the *A. baumannii* isolates (S1)*.* The results demonstrated that phage was specific for the *A. baumannii* and did not affect *Klebsiella, Pseudomonas*, *E. coli.*

### Bacterial reduction assay

**Fig. 10:**
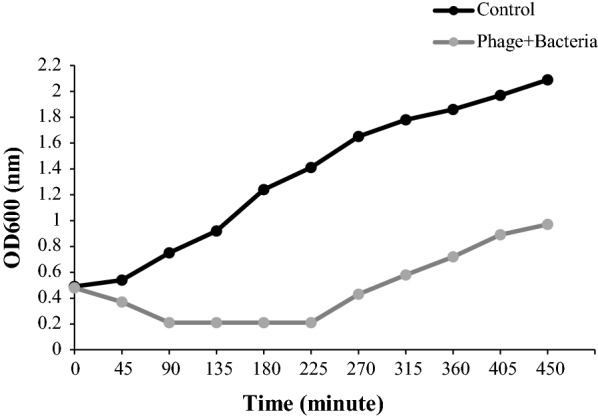
Bacterial reduction assay. Effect of pIsfAB02 on the growth of MDR-AB02 compared with the control

Infections of *A. baumannii* with a high titer of the lysate (10^10^ PFU/ml) were monitored for 7.5 h. Phage infection significantly decreased the *A. baumannii* culture turbidity in comparison to control. However, an increase in turbidity (OD_600_) was observed after about 4 h of culture incubation. This increase in turbidity was most probably due to the growth of phage-resistant bacteria (Fig. 10).

### Cell survival assay under bacterial infection

**Fig. 11:**
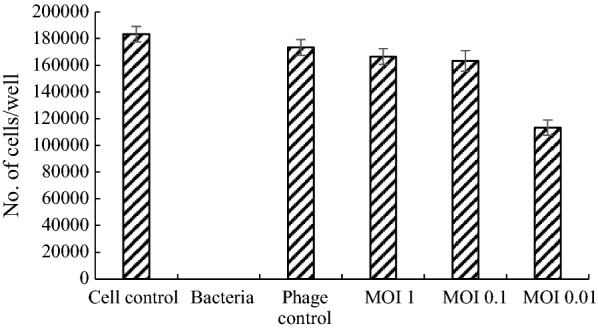
The cell survival assay of pIsf-AB02 on HeLa cells. The cells were infected with 10^6^ CFU of MDR-AB02. Phage at different MOI (10, 1, 0.1, 0.01) was added to the wells. The cell control; Hela cell, Phage control: phage with MOI 10 without bacteria. MOI 1, 0.1, and 0.01 of phage with exposure of *A. baumannii*

For the safety of phage therapy, HeLa cells were used to examine the cytotoxicity of the isolated phage in the presence of MDR-AB02. In 96-wells plate, different dilutions MOI = 10, 1, 0.1, 0.01 of pIsf-AB02 was added in the presence of 10^6^ CFU/ml MDR-AB02. The phage showed the highest protection against *A. baumannii* AB02 infection of cells (10^4^ cells/well) (Fig. 11). Phage at an MOI of 10, 1, 0.1 enabled cells inoculated with A. baumannii AB02 (10^6^ CFU) to survive as well as uninoculated controls. Although, the lowest cell viability was found at an MOI of 0.01. In bacterial control (cells inoculated with MDR-AB02 without phage treatment), all cells were completely killed by *A. baumannii*. Cells treated with the phage at an MOI of 10 (10^7^ PFU), but not inoculated with bacteria, survived as well as the control cells (p > 0.05), indicating that high dose of pIsf-AB02 did not affect HeLa survival. The results showed that pIsf-AB02 eliminated bacteria and protected HeLa cells from immediate killing by *A. baumannii* AB02 bacteria.

**Fig. 12:**
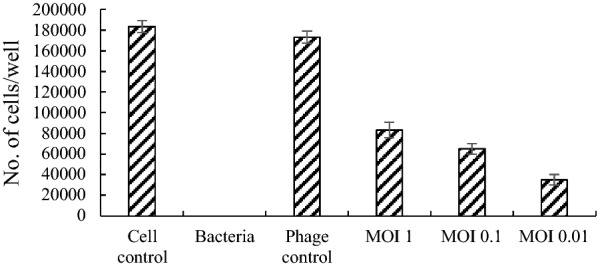
Protection efficacy test of pISF-AB02 on Hela Cell 2 h post infection: The cells (0.05 × 10^4^ cells/well) were first infected with 10^6^ CFU of MDR-AB02, and the Hela cells were counted after 12 h

In another experiment, the cell viability was assessed by adding the phage 2 h post-infection of the cells with MDR-AB02. As shown in Fig. 12, the pIsf-AB02 could not protect the cells at different MOIs (p < 0.001).

## Discussion

The objective of our study was to demonstrate a new approach for using the bacteriophage to treat life-threatening MDR *A. baumannii* infections. The antibiotic sensitivity of 48 *A. baumannii* isolates revealed that the prevalence of MDR and pan-drug *A. baumannii* is increasing in the region, which is in agreement with other studies in different countries [31, 32].

We isolated and characterized pIsf-AB02, a lytic phage from a clinical MDR *A. baumannii* isolate. It was able to form clear pinpointed plaques on MDR-AB02 lawns, indicative of strong lytic activity, and a wider host range among MDR *A. baumannii* clinical isolates (27/48, 56.2%) than our previously screened bacteriophages [33]*.* The major limitations of phage therapy are their narrow host range and the emergence of bacteriophage-insensitive mutants (BIMs); therefore, a large cocktail of phages is needed to improve therapy by extending the host range and reducing resistance and it is very important to isolate novel phages to enrich the phage supply.

Studying the isolated phage by TEM indicated that phage pIsf-AB02 should be assigned to the family of *Myoviridae* based on its morphological characteristics. Regeimbal et al. in the United States also isolated a particular *A. baumannii* phage from sewage and stated that the phage had a shaft-shaped head along with a contractile tail and belonged to the *Myoviridae* family [33]. Our isolated phage was a 6-cupsid, fractured, and constricted phage in which the morphological results overlapped with the other investigations [23, 34].

In a study by Kusradze et al., an *A. baumannii* phage from sewage was isolated and introduced through microscopic examination to the *Myoviridae* family. They also examined the phage stability at different temperatures, pH, and chloroform and showed that after a 24-h incubation of the phage at 37 °C, the potency of the phage remained unchanged and was stable in exposure to chloroform and normal pH [23]. Broadly speaking, high pH stability and high thermal resistance made the phage remarkably pledged for practical usage in the deracination of *A. baumannii* contaminations and or the treatment of *A. baumannii* infections. Phage pIsf-AB02 revealed impressive characteristics compared to the other phages. After a 24-h inoculation, the phage exhibited a steady state for chloroform. These results are consistent with the results of Kusradze [23]. Thermal resistant phages were usually isolated from extreme thermal habits [35, 36]; however, they could also be found in other environments. Recently, the thermal resistant phages have been isolated and characterized from various dairy products [37, 38].

One-step growth curve analysis revealed a 30-min latent period, a 70-min lysis period, and a burst size of 120 phage particles per infected host cell. Compared with the isolated phage by Yang et al., pIsf-AB02 had a smaller burst size but a wider host range among the local *A. baumannii* isolates and a boarder range of temperature and pH stability [39], making pIsf-AB02 a suitable nominee for further application of phage therapy.

In 30 min, pIsf-AB02 with MOI of 1 reduced MDR-AB02 from 10^8^ CFU/ml to 10^3^ CFU/ml and maintained this concentration for about 2 h. However, the lower MOIs (0.1 and 0.01) decreased the virus titer to the same point in 1.5–2 h. The optimal titer of the phage in phage therapy is crucial. Despite the thought that the higher MOI of phage inoculation would get higher efficiency on waning the bacterium, the interaction between hosts and phages should be optimized. It might be possible that the high titer phage would occupy the receptor for phage and the bacterial lysis rate would not rise with an increase in MOI of phage [40]. Another possibility is that sometimes the high titer phage would induce the host immune system and limit phage therapy [41].

Herein, a high concentration of bacteria was applied to infect the HeLa cells to examine the protective efficacy of phages on the cells. The data showed that in the presence of the bacteria, pIsf-AB02 phage in higher MOIs didn’t affect on the survival rate significantly after a 24-h incubation in comparison to the control. On the other hand, the phage pIsf-AB02 did not have an adverse effect on the growth of HeLa cells, indicating that pIsf-AB02 is a suitable candidate for phage therapy. Further animal model experiments should be performed to confirm that phage pIsf-AB02 has a good protection on the animals infected by MDR *A. baumannii*.

Most double-stranded DNA phages accomplish host cell lysis through the holin-endolysin system. The similarity of bacteriophage endolysin genes is essential for structural analysis, which contributes to the potential of utilizing endolysin as an antimicrobial agent [42]. The endolysins antibacterial activity is generally attributed to their enzymatic function, which ruptures the covalent bonds in peptidoglycan. However, some endolysins, especially those from phages of Gram-negative bacteria, can affect the bacterial cells employing a mechanism completely independent of their enzymatic activity [43–45]. In the current work, the protein causing lysis was estimated to be about 15 kDa through SDS PAGE, which corroborated the previous results [46]. The lysin was utterly stable and constant over a wide range of pHs.

Phage candidates for therapeutic application purposes should not harbor foreign genes, such as virulence or antibiotic resistance genes, integrases, site-specific recombinases, and repressors of the lytic cycle [47]. Phages could serve as a vector for horizontal transfer virulence gene to bacteria, making them more pathogenic or resistant to antibiotics [48].Therefore, the genomic characterization of pIsf-AB02 is very important and should be taken into consideration to guarantee the safety of phage therapy. Phages may show synergistic effects when combined with antibiotics [49, 50]. A reduction in the formation of bacterial biofilms has also been reported when antibiotic treatment is applied in combination with phages [51, 52]. These conclusions should be verified through future studies to further overcome the limitations of phage therapy.

In this study, we isolated and characterized a novel lytic *A. baumannii* bacteriophage and evaluated the lytic activity of the phage against the isolated MDR *A. baumannii*. Furthermore, we assessed the efficacy of phage endolysin on MDR *A. baumannii* clinical isolates. Our findings support the potential application of the phage with the potent endolysin activity against MDR *A. baumannii* and suggest that this phage could be developed for the treatment of MDR *A. baumannii* infections.

## Conclusion

In this study, we isolated a novel lytic MDR *A. baumannii* bacteriophage, pIsf-AB02. This phage showed suitable stability at different temperatures and pHs, and demonstrated potent in vitro endolysin activity. pIsf-AB02 may be a good candidate as a therapeutic agent to control nosocomial infections caused by MDR *A. baumannii*.

## Supplementary Information

**Additional file 1: Table S1. ** The antibiotic sensitivity results and the spot test of pISF-AB2 phage, **Figure S1.** pH stability test, Figure S2: Thermal stability of pISF-AB2 phage.

## Data Availability

The datasets used and/or analyzed during the current study are available from the corresponding author on reasonable request.

## Abbreviations

MDR: Multidrug resistance
ICU: Intensive Care Units
ESKAPE: (*Enterococcus faecium, Staphylococcus aureus*, *Klebsiella pneumonia*, *Acinetobacter baumannii*, *Pseudomonas aeruginosa* And* Enterobacter *spp)
MOI: Multiplicity of infection
OD: Optimal density
PFU: Plaque-forming unit
CFU: Colony-forming unit
pH: The potential of hydrogen
TEM: Transmission electron microscopy
NB: Nutrient broth
TBE: Tris-boric acid-EDTA
ATCC: American type culture collection
