# Supplementary material for: The characteristic and potential therapeutic effect of isolated multidrug-resistant Acinetobacter baumannii lytic phage
Source: Ann Clin Microbiol Antimicrob. 2022 Jan 7;21:1. doi: 10.1186/s12941-022-00492-9 (PMC8742398; doi:10.1186/s12941-022-00492-9)
Supplement: Supplementary file 1 — Additional file 1: Table S1. The antibiotic sensitivity results and the spot test of pISF-AB2 phage, Figure S1. pH stability test, Figure S2: Thermal stability of pISF-AB2 phage. [file 12941_2022_492_MOESM1_ESM.docx]

| Isolates | Source | Phage  (Isf-AB2) | Antibiotic sensitivity | | | | | |
| --- | --- | --- | --- | --- | --- | --- | --- | --- |
|  |  |  | **Imipenem** | **Ciprofloxacin** | **Ceftazidime** | **Cefepime** | **Rifampin** | Amikacin |
| AB01 | Blood | - | R | R | R | S | S | R |
| AB02 | Catheter | + | R | R | R | R | R | R |
| AB03 | Catheter | + | R | R | R | R | S | S |
| AB04 | Catheter | + | R | R | R | R | R | R |
| AB05 | Catheter | + | R | R | R | R | R | R |
| AB06 | Catheter | - | R | R | R | R | R | R |
| AB07 | Wound | - | R | R | R | R | S | R |
| AB08 | Blood | + | R | R | R | R | R | R |
| AB09 | Catheter | + | R | R | R | R | R | S |
| AB 11 | Wound | - | R | R | R | R | R | R |
| AB 11 | Wound | + | R | R | R | R | R | S |
| AB 12 | Catheter | - | R | R | S | R | R | R |
| AB 13 | Catheter | + | R | R | R | R | R | R |
| AB 14 | Urine | + | R | R | R | S | R | R |
| AB 15 | Blood | + | R | R | R | R | R | R |
| AB 16 | Blood | - | R | R | R | R | R | R |
| AB 17 | Catheter | + | R | R | R | R | R | R |
| AB 18 | Catheter | - | R | R | R | R | R | R |
| AB 19 | Catheter | + | R | R | R | R | R | R |
| AB 20 | Urine | - | R | R | R | R | R | R |
| AB 21 | Wound | - | R | R | R | R | R | S |
| AB 22 | Urine | + | R | R | R | R | S | R |
| AB 23 | CSF | + | R | R | R | R | R | R |
| AB 24 | Blood | - | R | S | R | R | S | R |
| AB 25 | Urine | + | R | R | R | R | R | R |
| AB 26 | Wound | - | R | R | R | R | R | R |
| AB 27 | Catheter | + | R | R | R | R | R | R |
| AB 28 | Catheter | + | R | R | R | R | R | S |
| AB 29 | CSF | - | R | R | R | R | R | R |
| AB 31 | Sputum | + | R | R | R | R | R | S |
| AB 31 | Blood | + | R | R | R | R | R | R |
| AB 32 | Wound | - | R | R | R | R | R | R |
| AB 33 | Urine | - | R | R | R | R | R | R |
| AB 34 | Sputum | - | R | R | R | R | R | S |
| AB 35 | Urine | + | R | R | R | R | S | R |
| AB 36 | Blood | - | R | R | R | R | R | R |
| AB 37 | Catheter | + | R | R | R | R | S | S |
| AB 38 | Wound | - | R | R | R | R | R | R |
| AB 39 | Catheter | + | R | R | R | R | R | R |
| AB 41 | Urine | + | R | R | R | R | R | R |
| AB 41 | Catheter | - | R | R | R | R | R | R |
| AB 42 | Catheter | + | R | R | R | R | R | R |
| AB 43 | Blood | + | R | R | R | R | R | R |
| AB 44 | Wound | - | R | R | R | R | R | R |
| AB 45 | Wound | - | R | R | R | R | R | R |
| AB 46 | Catheter | + | R | R | R | R | R | S |
| AB 47 | Catheter | - | R | R | R | R | R | R |
| AB 48 | Urine | + | R | R | S | R | R | R |

Table S1. The antibiotic sensitivity results and the spot test of pISF-AB2 phage against *A. baumannii* isolates used in this study.

Figure S1. pH stability of AB- phage at different pH

Figure S2: Thermal stability of phage under different temperatures
